# Supplementary material for: Chemical reaction-mediated covalent localization of bacteria
Source: Nat Commun. 2022 Dec 17;13:7808. doi: 10.1038/s41467-022-35579-6 (PMC9759558; doi:10.1038/s41467-022-35579-6)
Supplement: Supplementary file 1 — Supplementary Information [file 41467_2022_35579_MOESM1_ESM.pdf]

Supplementary Information

Chemical reaction-mediated covalent localization of bacteria

*Huilong Luo[1], Yanmei Chen[1], Xiao Kuang[1], Xinyue Wang[1], Fengmin Yang[1], Zhenping Cao[1], Lu Wang[1], Sisi Lin[1], Feng Wu[1], Jinyao Liu[1]**

[1]Shanghai Key Laboratory for Nucleic Acid Chemistry and Nanomedicine, Institute of Molecular Medicine, State Key Laboratory of Oncogenes and Related Genes, Shanghai Cancer Institute, Renji Hospital, School of Medicine, Shanghai Jiao Tong University, Shanghai 200127, China.

*Correspondence: J.L. (jyliu@sjtu.edu.cn)

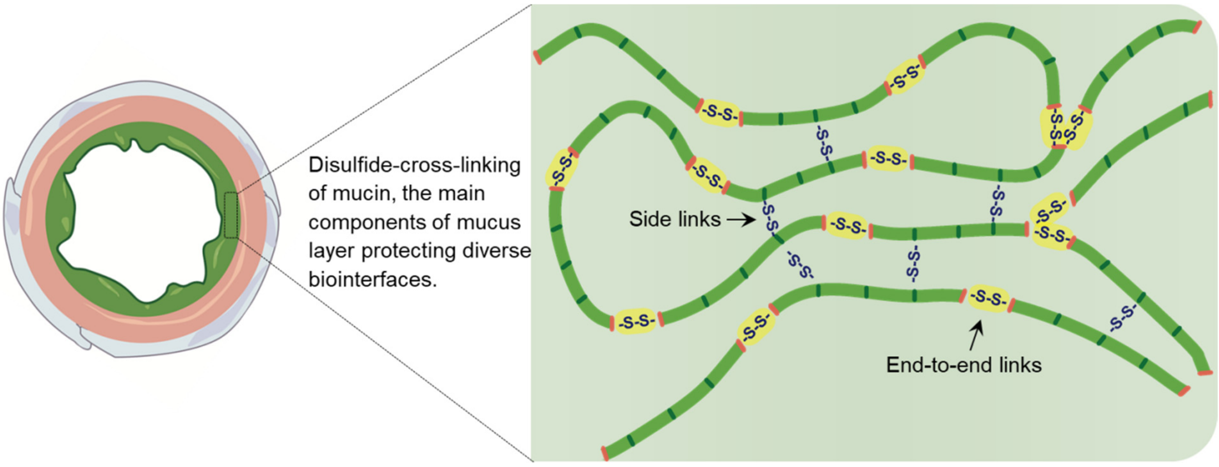

Supplementary Fig. 1. Structure of mucins. Mucins are an important category of heavily glycosylated proteins existing at various tissue interfaces, with cysteine-rich domains in their N and C termini that mediate polymer extension by end-to-end disulfide linkage of mucin monomers. Cysteine-rich regions locating in internal domains also contribute to disulfide side links between intermediate cysteine thiols.

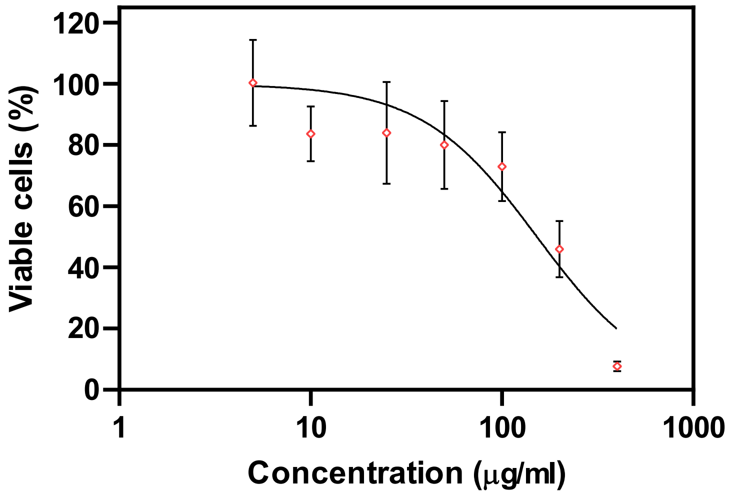

Supplementary Fig. 2. Representative curve of bacterial survival after reaction with 2-iminothiolane (IC_{50} 152.3 μg/ml). The number of viable cell was counted by bacterial plating counting. Data are presented as mean values ± SD (n = 3, from independent experiments). Source data are provided in the Source Data file.

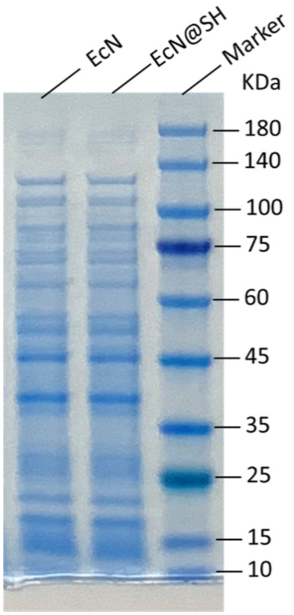

Supplementary Fig. 3. Coomassie blue staining of the total proteins extracted from native and thiolated EcN, respectively. Images are representative of three independent biological samples.

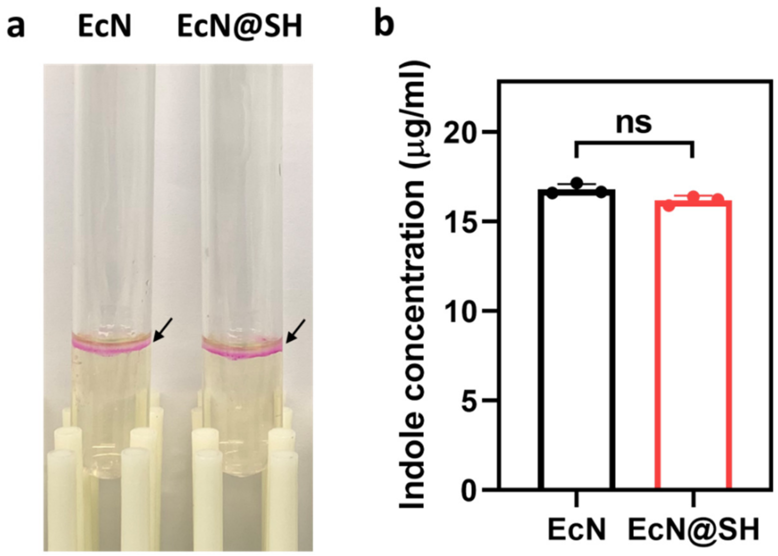

Supplementary Fig. 4. a Digital photos of the indole test on the tryptophan metabolism of native and thiolated EcN. Black arrows indicate the purple indole ring of positive results. **b** Quantitative detection of indole production by HPLC. Data are presented as mean values ± SD (n = 3, from independent biological samples). Significance was assessed using Student's t-test (two-tailed). ns, no significance. Source data are provided in the Source Data file.

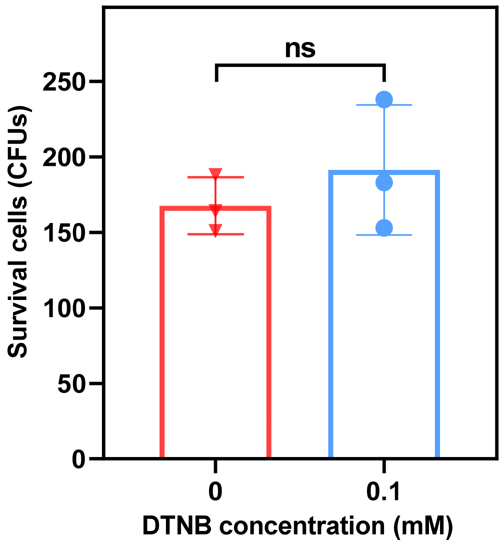

Supplementary Fig. 5. Survival cells after treatment with 0.1 mM DTNB for 2 hours. Data are presented as mean values ± SD (n = 3, from independent experiments). Significance was assessed using Student's t-test (two-tailed). ns, no significance. Source data are provided in the Source Data file.

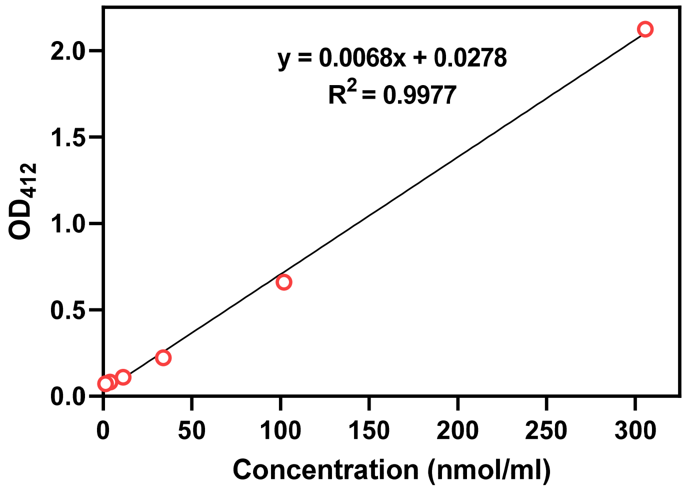

Supplementary Fig. 6. Standard curve for quantitatively measuring thiol content in varied concentrations of L-cysteine using Traut's reagent.

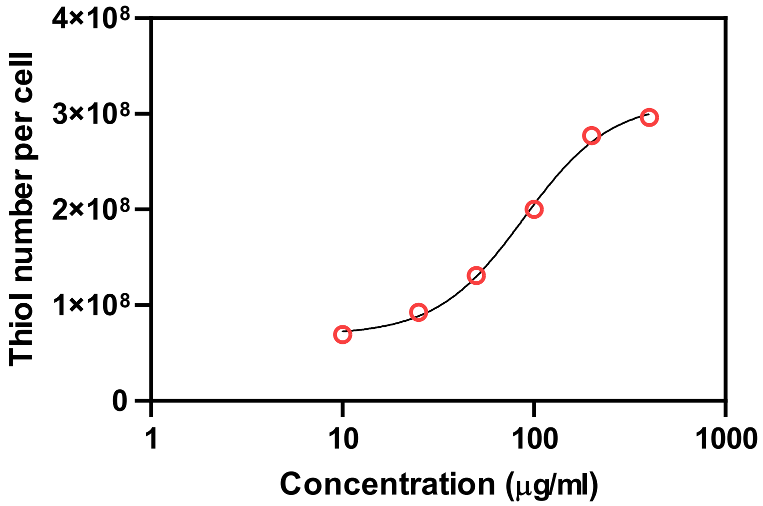

Supplementary Fig. 7. Representative curve of thiol number per bacterium after reaction with 2-iminothiolane ranging from 0 to 400 µg/mL for 90 minutes (EC_{50} 88.3 µg/ml). Source data are provided in the Source Data file.

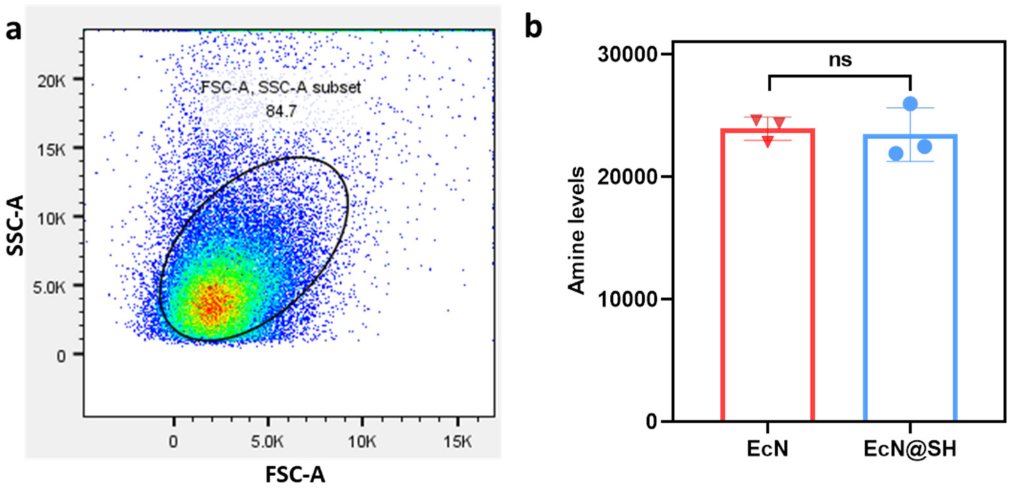

Supplementary Fig. 8. a Gate strategy to select bacterial cells for Cy5-NHS labelling using FSC-A and SSC-A. Dead cells were excluded and further analysis were performed on the live cell population. **b** Amine levels of native EcN and EcN@SH measured by flow cytometry using Cy5-NHS labelling. Data are presented as mean values ± SD (n = 3, from independent experiments). Significance was assessed using Student's t-test (two-tailed). ns, no significance. Source data are provided in the Source Data file.

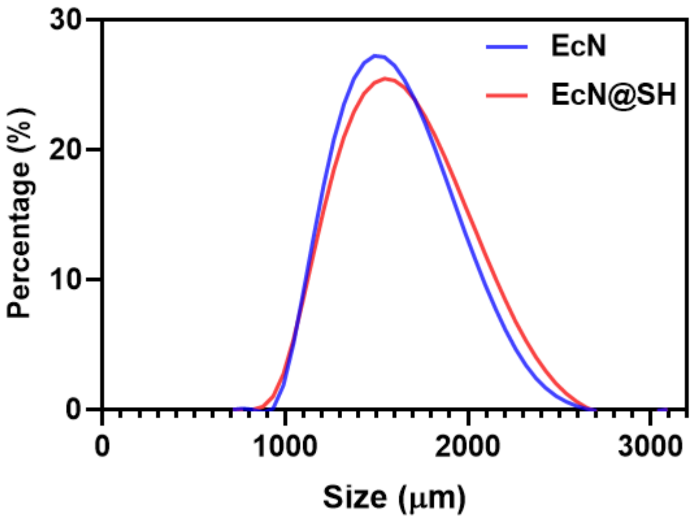

Supplementary Fig. 9. Size distributions of native EcN and EcN@SH measured by DLS. Source data are provided in the Source Data file.

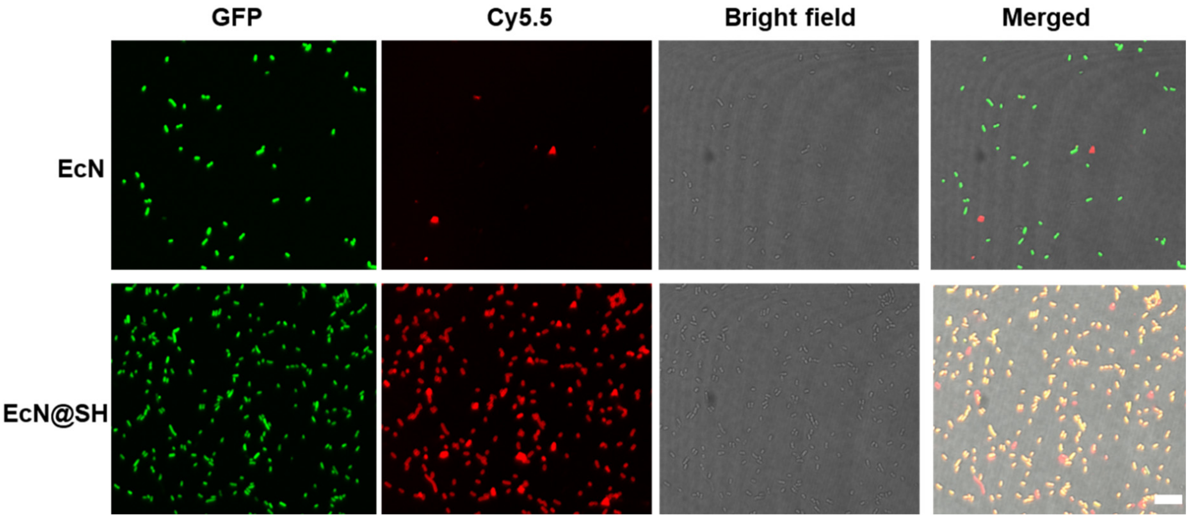

Supplementary Fig. 10. LSCM images of unmodified EcN and thiolated EcN@SH post labelling with Cy5.5-maleimide. Images are representative of three independent biological samples. Green and red channels separately show inherent GFP of EcN and Cy5.5-maleimide chemically-stained thiols layer. Scale bar: 10 μm.

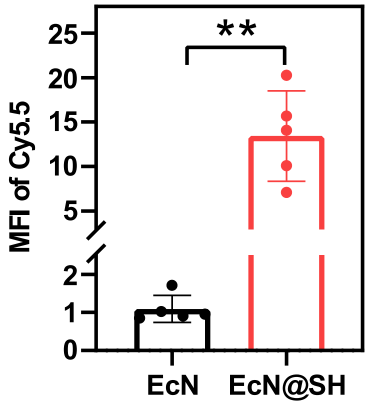

Supplementary Fig. 11. Mean fluorescence intensity (MFI) of Cy5.5-maleimide labelled thiol groups on EcN and EcN@SH by confocal analysis. Data are presented as mean values ± SD (*n* = 5, from independent fields). Significance was assessed using Student's *t*-test (two-tailed), giving *p* values, $^{**}p < 0.01$. Source data and the exact *p* values are provided in the Source Data file.

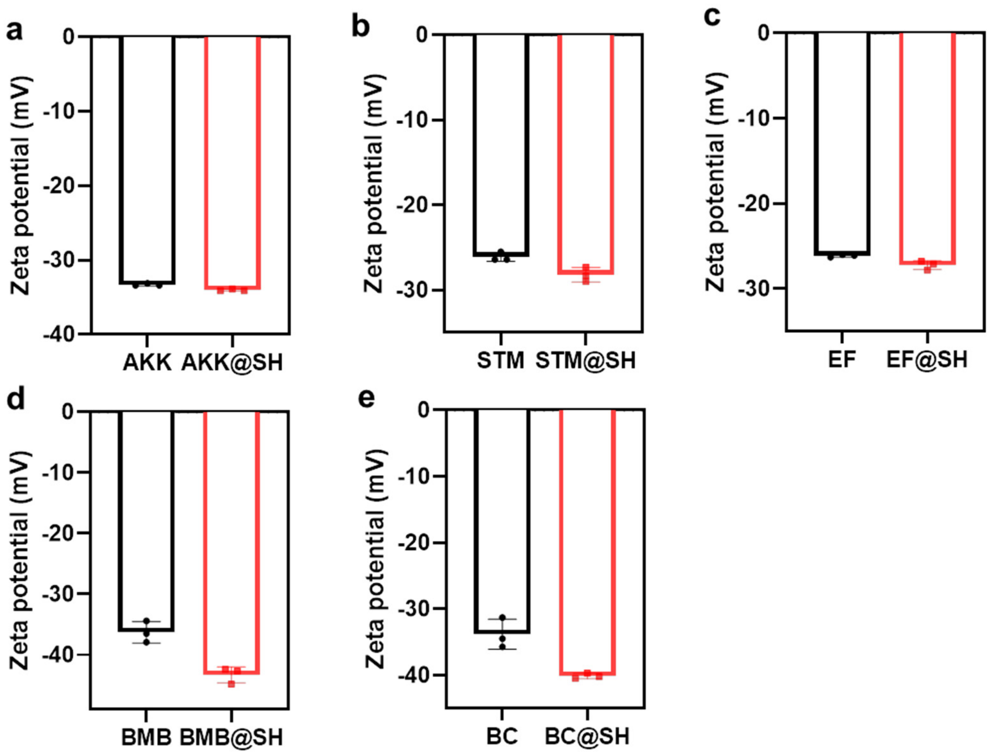

Supplementary Fig. 12. Zeta potentials of native and thiolated bacteria including (**a**) AKK, (**b**) STM, (**c**) EF, (**d**) BMB, and (**e**) BC. Data are presented as mean values ± SD (*n* = 3, from independent experiments). Source data are provided in the Source Data file.

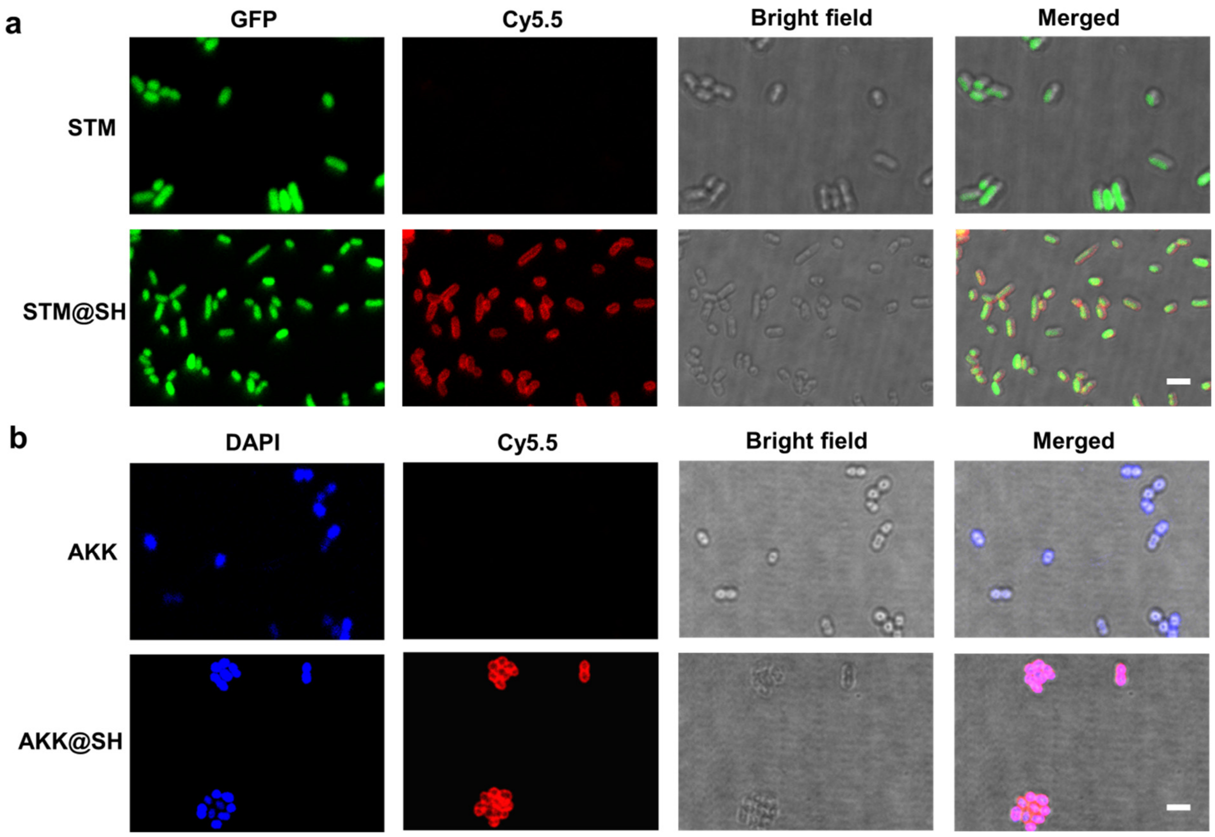

Supplementary Fig. 13. Typical LSCM images of unmodified and thiolated Gram negative bacteria after reaction with Cy5.5-maleimide, including **a** STM and **b** AKK. Images are representative of three independent biological samples. Scale bar: 2 μm.

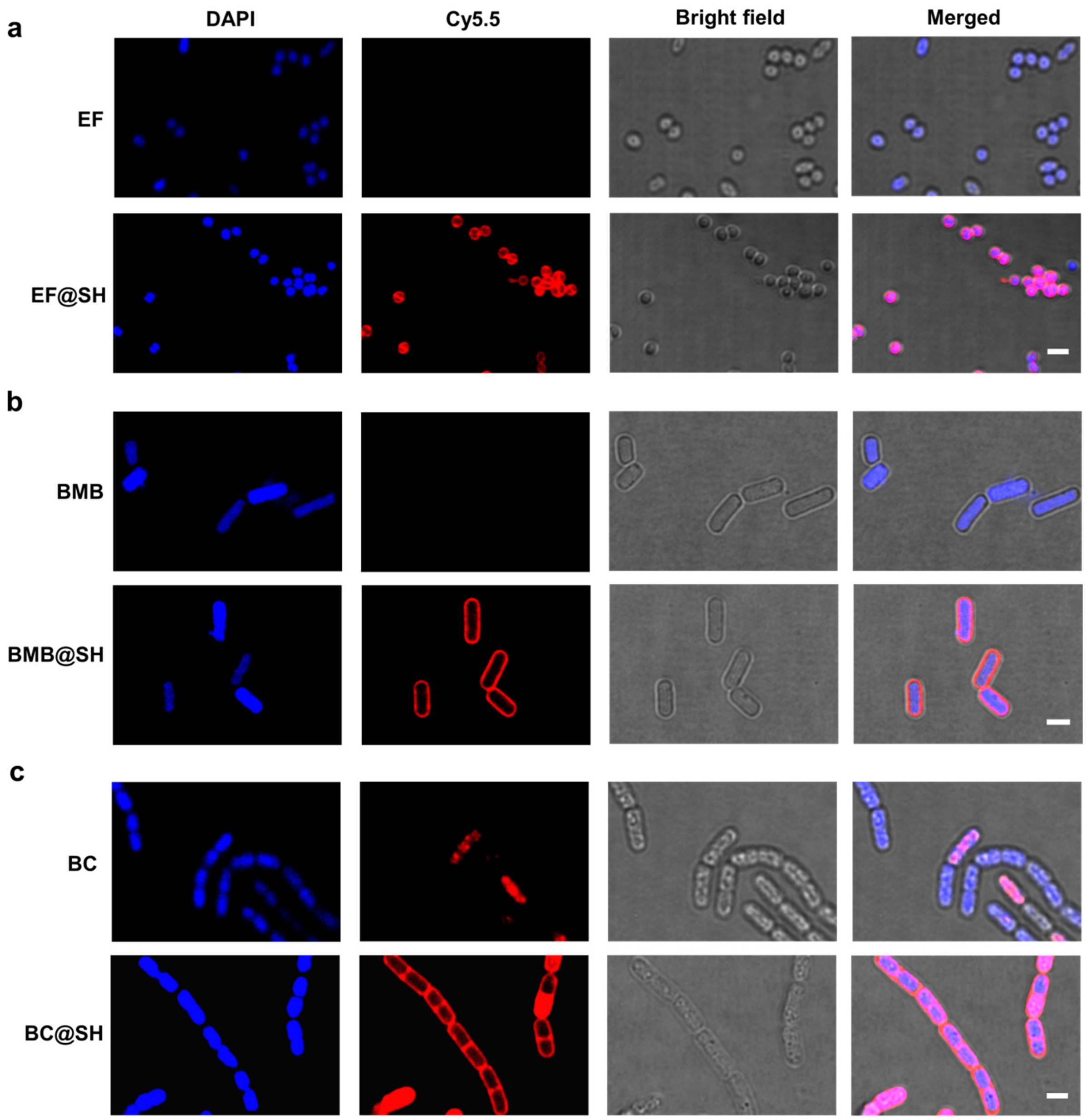

Supplementary Fig. 14. Typical LSCM images of unmodified and thiolated Gram positive bacteria after reaction with Cy5.5-maleimide, including **a** STM, **b** BMB, and **c** BC. Images are representative of three independent biological samples. Scale bar: 2 μm.

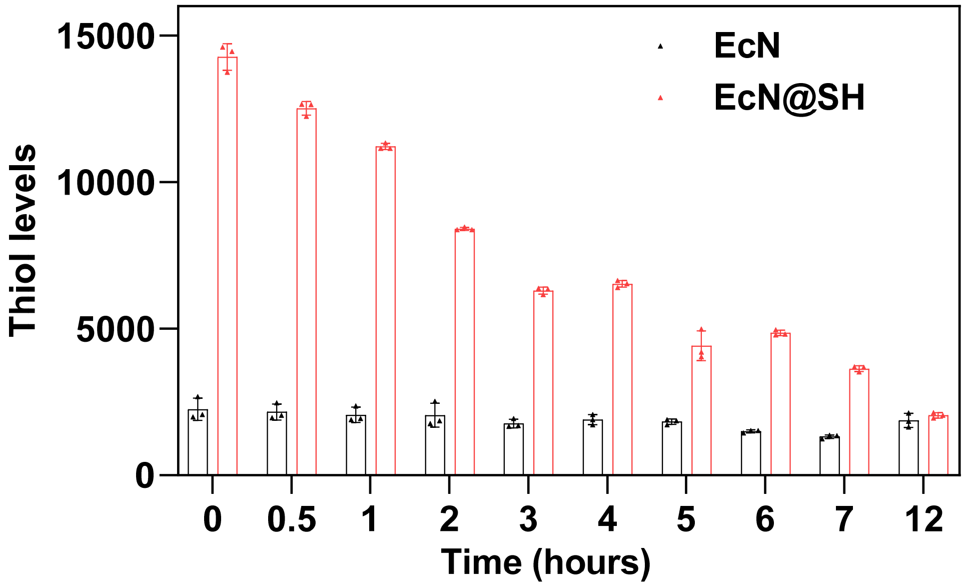

Supplementary Fig. 15. Retention of thiol levels on native EcN and EcN@SH over time measured by flow cytometry using SH-sensitive and clickable marker Cy5.5-maleimide. Data are presented as mean values ± SD (n = 3, from independent experiments). Source data are provided in the Source Data file.

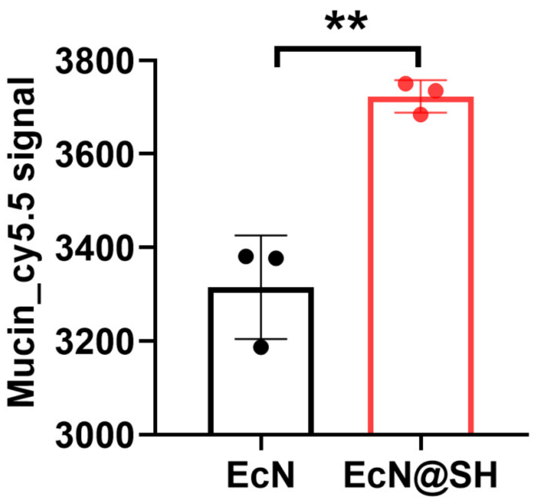

Supplementary Fig. 16. Flow cytometric analysis of native EcN and EcN@SH after reaction with Cy5.5_mucin (0.1 mg/ml) for 1 hour. Data are presented as mean values ± SD (n = 3, from independent experiments). Significance was assessed using Student's t-test (two-tailed), giving p values, **$p < 0.01$. Source data and the exact p values are provided in the Source Data file.

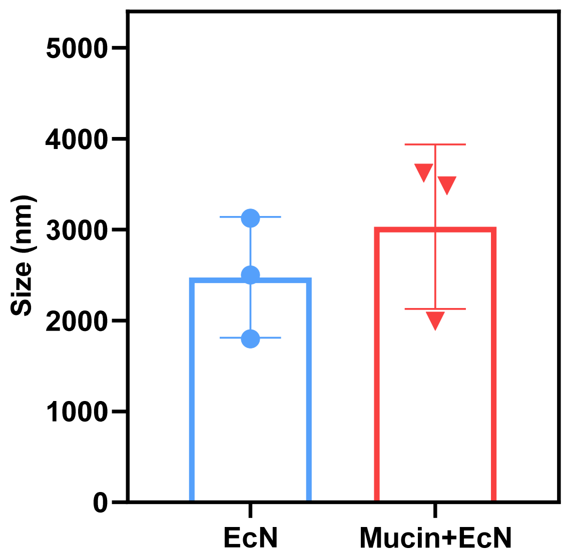

Supplementary Fig. 17. Sizes of EcN and mucin-attached EcN measured by DLS. Data are presented as mean values ± SD (n = 3, from independent experiments). Source data are provided in the Source Data file.

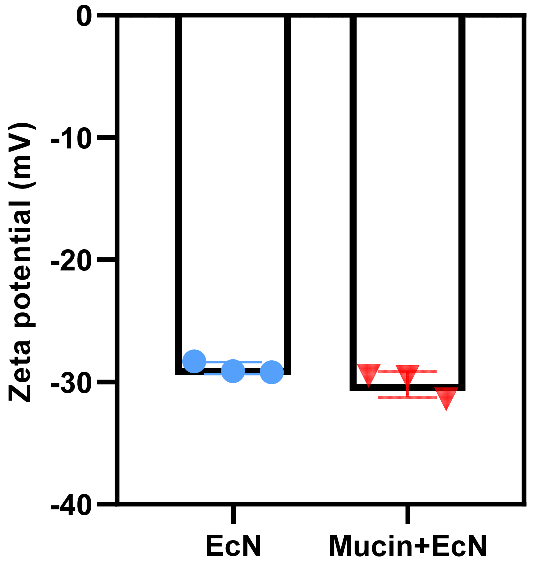

Supplementary Fig. 18. Zeta potentials of EcN and mucin-attached EcN measured by DLS. Data are presented as mean values ± SD (n = 3, from independent experiments). Source data are provided in the Source Data file.

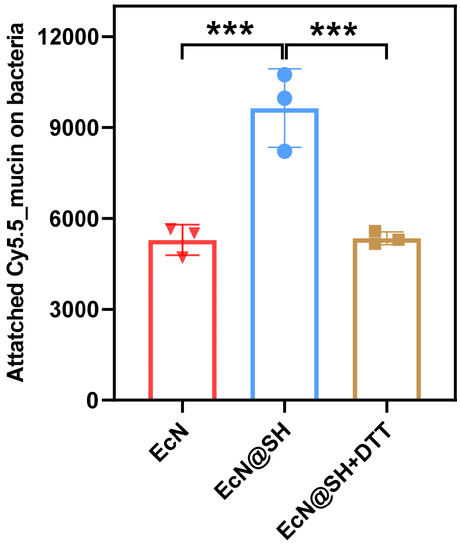

Supplementary Fig. 19. MFI values of EcN@SH after reaction with 0.15 mg/ml of Cy5.5_mucin in the presence of 0. 1 mg/ml of DTT. Data are presented as mean values ± SD (n = 3, from independent experiments). Significance was assessed using one-way ANOVA analysis followed by Fisher's LSD multiple comparisons, giving p values, ***$p < 0.001$. Source data and the exact p values are provided in the Source Data file.

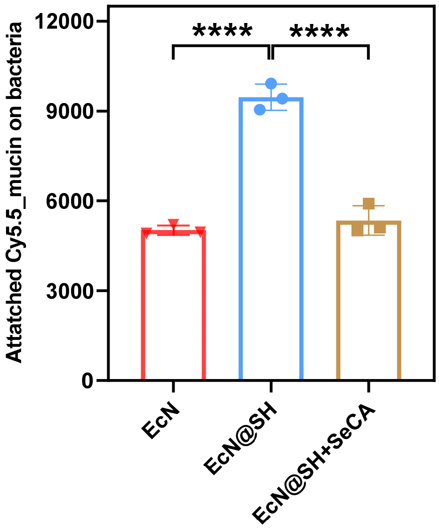

Supplementary Fig. 20. MFI values of EcN@SH after reaction with 0.15 mg/ml of Cy5.5_mucin in the presence of 0.3 mg/ml of SeCA. Data are presented as mean values ± SD (n = 3, from independent experiments). Significance was assessed using one-way ANOVA analysis followed by Fisher's LSD multiple comparisons, giving p values, ****$p < 0.0001$. Source data and the exact p values are provided in the Source Data file.

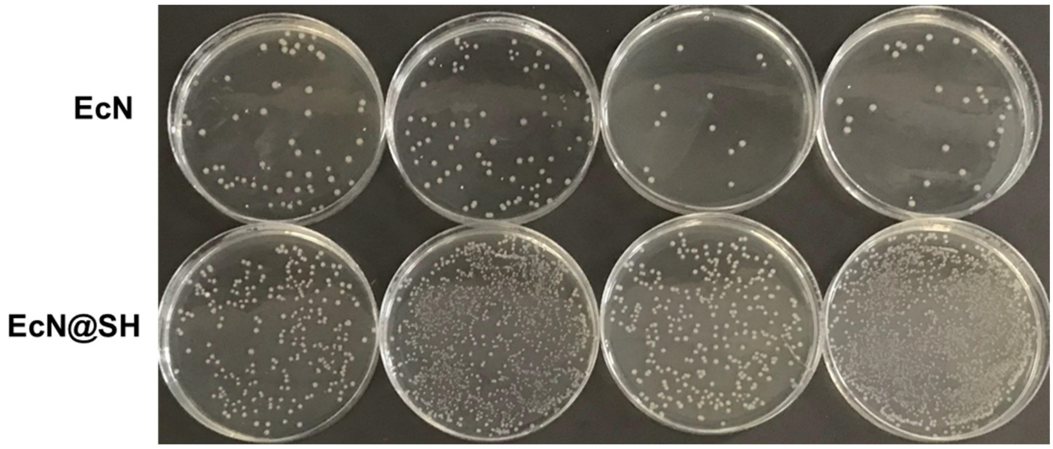

Supplementary Fig. 21. Representative images of agar plates containing EcN collected from jejunal mucus 4 hours after oral administration of equivalent EcN and EcN@SH (1.0×10^8 CFUs per mouse).

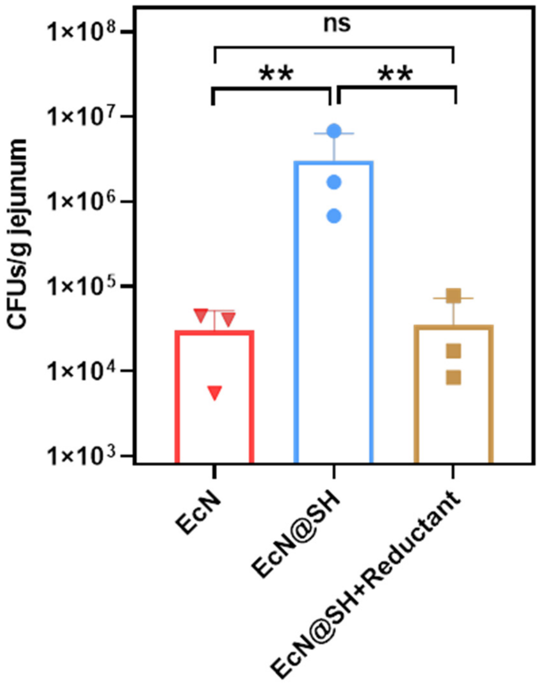

Supplementary Fig. 22. Counts of EcN collected from mouse jejunal mucus at 1 hour after oral delivery of unmodified EcN, EcN@SH, or EcN@SH plus 1 mg/ml of L-ascorbic acid. Data are presented as mean values ± SD (n = 3 mice). Significance was assessed using one-way ANOVA analysis followed by Fisher's LSD multiple comparisons, giving p values, $^{**}p < 0.01$. ns, no significance. Source data and the exact p values are provided in the Source Data file.

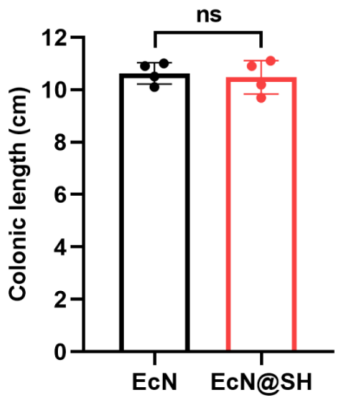

Supplementary Fig. 23. Average intestinal length after administration with equivalent EcN or EcN@SH (1.0×10^8 CFUs per mouse). Data are presented as mean values ± SD (n = 4 mice). Significance was assessed using Student's *t*-test (two-tailed). ns, no significance. Source data are provided in the Source Data file.

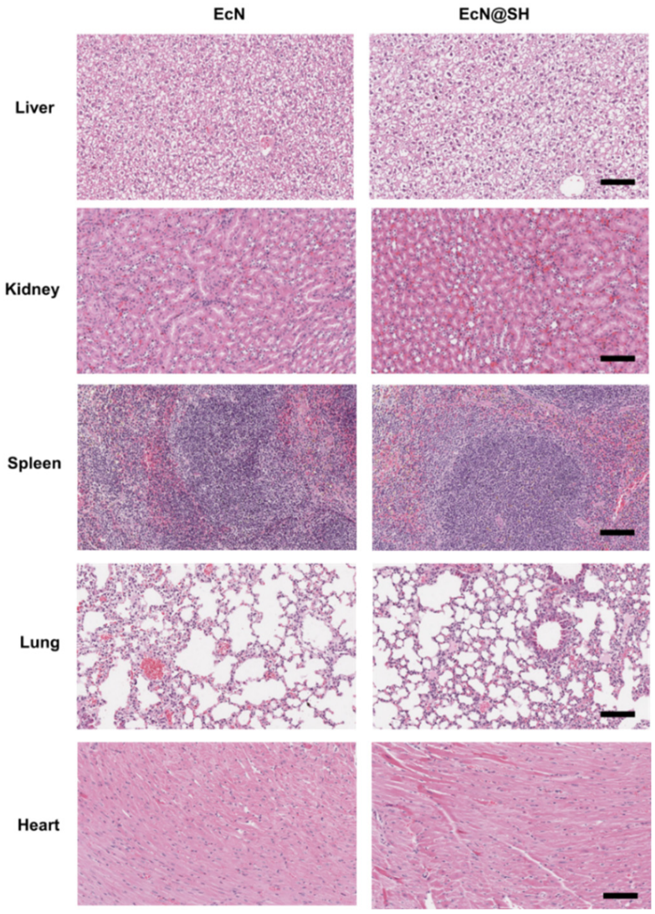

Supplementary Fig. 24. H&E staining images of liver, kidney, spleen, lung, and heart tissues sectioned from mice administered with equivalent unmodified EcN or EcN@SH (1.0×10^8 CFUs per mouse). Images are representative of four independent biological samples. Scale bar: 100 μm.

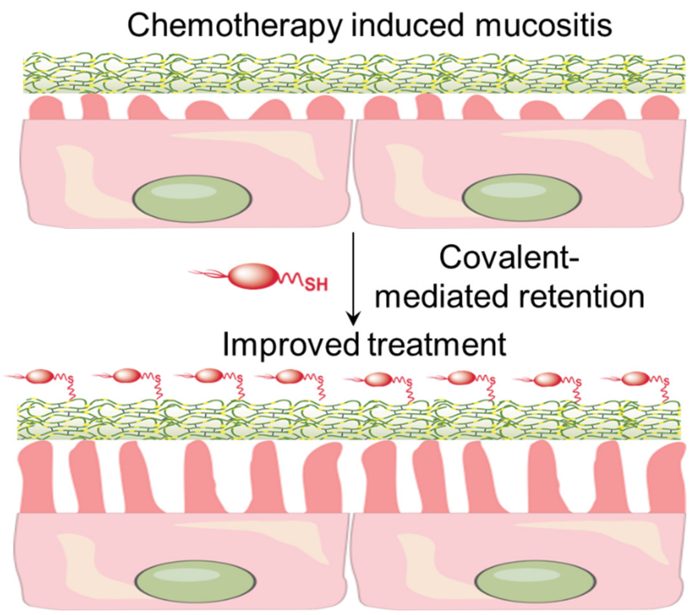

Supplementary Fig. 25. Schematic illustration of enhanced treatment of jejunal mucositis by covalent bonding mediated localization of thiolated EcN.

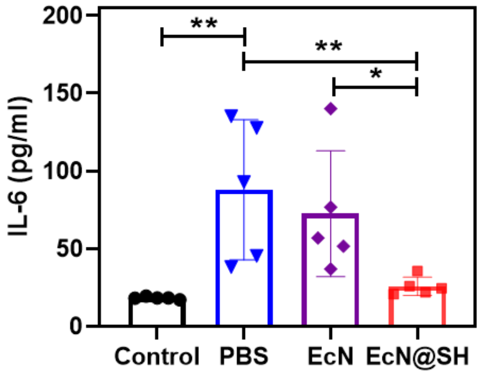

Supplementary Fig. 26. Level of IL-6 in serum measured by commercially available ELISA kits. Data are presented as mean values ± SD (n = 5 mice). Significance was assessed using one-way ANOVA analysis followed by Fisher's LSD multiple comparisons, giving p values, $*p < 0.05$, $**p < 0.01$. Source data and the exact p values are provided in the Source Data file.

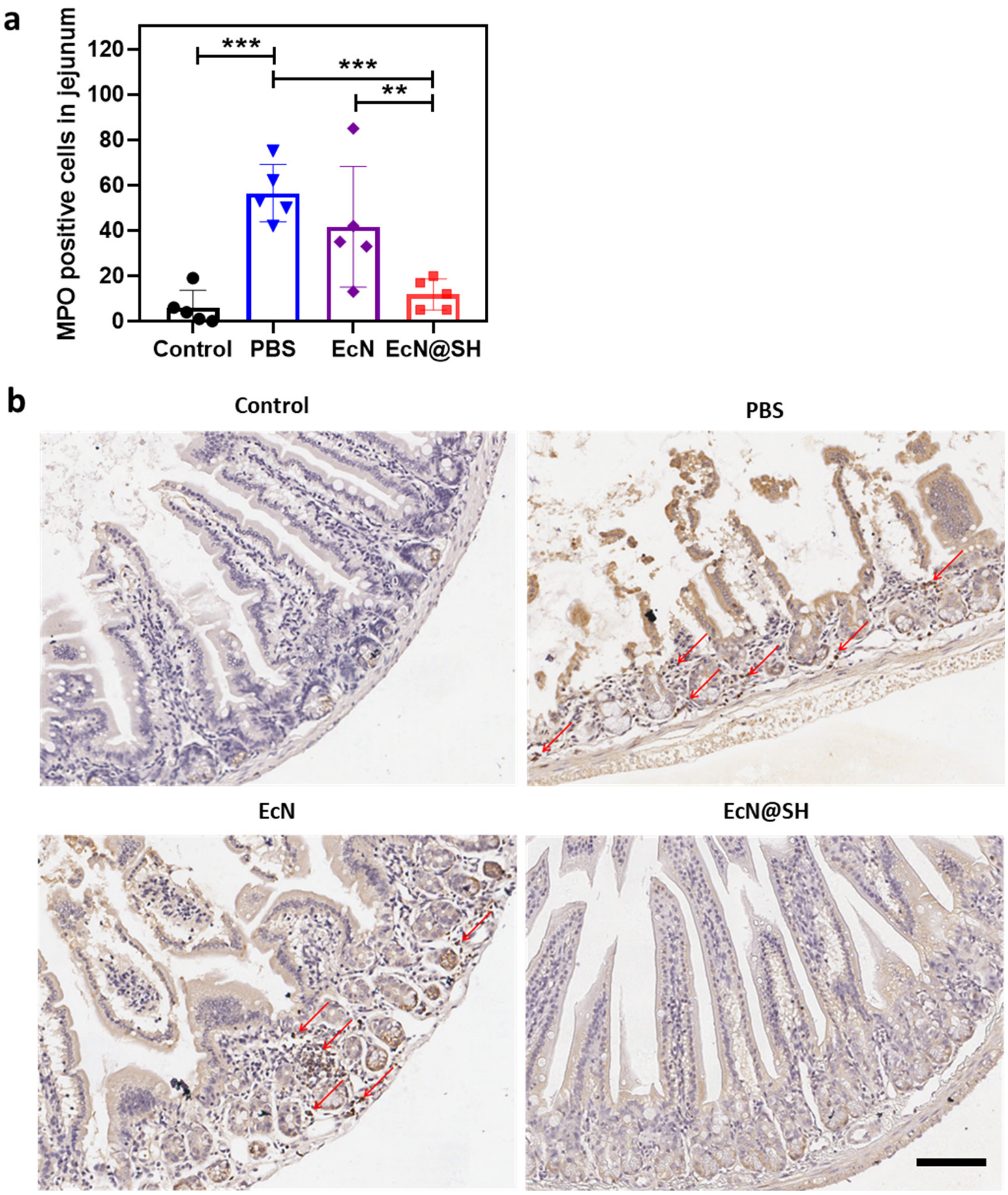

Supplementary Fig. 27. a Mean MPO positive cells counted in jejunm. **b** Typical images of MPO staining of jejunum tissues. Red arrows indicate MPO positive cells. Scale bar: 50 µm. Data are presented as mean values ± SD (*n* = 5 mice). Significance was assessed using one-way ANOVA analysis followed by Fisher's LSD multiple comparisons, giving *p* values, ***p* < 0.01, ****p* < 0.001. Source data and the exact *p* values are provided in the Source Data file.
