# Supplementary material for: Chemical reaction-mediated covalent localization of bacteria
Source: Nat Commun. 2022 Dec 17;13:7808. doi: 10.1038/s41467-022-35579-6 (PMC9759558; doi:10.1038/s41467-022-35579-6)
Supplement: Supplementary file 3 — Reporting Summary [file 41467_2022_35579_MOESM3_ESM.pdf]

nature portfolio

Corresponding author(s): Jinyao Liu

Last updated by author(s): Dec 3, 2022

Reporting Summary

Nature Portfolio wishes to improve the reproducibility of the work that we publish. This form provides structure for consistency and transparency in reporting. For further information on Nature Portfolio policies, see our Editorial Policies and the Editorial Policy Checklist.

Statistics

For all statistical analyses, confirm that the following items are present in the figure legend, table legend, main text, or Methods section.

n/a	Confirmed	
☐	☒	The exact sample size (*n*) for each experimental group/condition, given as a discrete number and unit of measurement
☐	☒	A statement on whether measurements were taken from distinct samples or whether the same sample was measured repeatedly
☐	☒	The statistical test(s) used AND whether they are one- or two-sided<br>*Only common tests should be described solely by name; describe more complex techniques in the Methods section.*
☐	☒	A description of all covariates tested
☐	☒	A description of any assumptions or corrections, such as tests of normality and adjustment for multiple comparisons
☐	☒	A full description of the statistical parameters including central tendency (e.g. means) or other basic estimates (e.g. regression coefficient) AND variation (e.g. standard deviation) or associated estimates of uncertainty (e.g. confidence intervals)
☐	☒	For null hypothesis testing, the test statistic (e.g. *F*, *t*, *r*) with confidence intervals, effect sizes, degrees of freedom and *P* value noted<br>*Give P values as exact values whenever suitable.*
☒	☐	For Bayesian analysis, information on the choice of priors and Markov chain Monte Carlo settings
☒	☐	For hierarchical and complex designs, identification of the appropriate level for tests and full reporting of outcomes
☒	☐	Estimates of effect sizes (e.g. Cohen's *d*, Pearson's *r*), indicating how they were calculated

Our web collection on statistics for biologists contains articles on many of the points above.

Software and code

Policy information about availability of computer code

Data collection	Flow cytometry (CytoFLEX supplemented with CytExpert system, Beckman, Coulter, USA); Confocal microscopy (Leica TCS SP8, Germany)
Data analysis	Statistical analysis was performed using GraphPad Prism 8 version 8.0.1. The Flow cytometry data were analyzed by Flowjo v10. The images were analyzed by ImageJ 1.53k software.

For manuscripts utilizing custom algorithms or software that are central to the research but not yet described in published literature, software must be made available to editors and reviewers. We strongly encourage code deposition in a community repository (e.g. GitHub). See the Nature Portfolio guidelines for submitting code & software for further information.

Data

Policy information about availability of data

All manuscripts must include a data availability statement. This statement should provide the following information, where applicable:

- Accession codes, unique identifiers, or web links for publicly available datasets
- A description of any restrictions on data availability
- For clinical datasets or third party data, please ensure that the statement adheres to our policy

All data are available within the article or Supplementary information. Source data are provided with this paper.

Human research participants

Policy information about studies involving human research participants and Sex and Gender in Research.

Reporting on sex and gender	The study did not involve human research. Not Applicable.
Population characteristics	Not Applicable.
Recruitment	Not Applicable.
Ethics oversight	Not Applicable.

Note that full information on the approval of the study protocol must also be provided in the manuscript.

Field-specific reporting

Please select the one below that is the best fit for your research. If you are not sure, read the appropriate sections before making your selection.

☒ Life sciences ☐ Behavioural & social sciences ☐ Ecological, evolutionary & environmental sciences

For a reference copy of the document with all sections, see nature.com/documents/nr-reporting-summary-flat.pdf

Life sciences study design

All studies must disclose on these points even when the disclosure is negative.

Sample size	Sample sizes were determined to allow the statistical significance of differences of 50% or greater, and according to similar studies conducted in the field (Cao et al, Nature Communications 2019, 10:5783; Pan et al, Advanced Materials, 2021, 2007379). Sample size and the number of biological replicates for each experiment are indicated in the figure legends.
Data exclusions	No data was excluded.
Replication	All experiments were conducted at least two times and could be reliably reproduced.
Randomization	Within each experimental regime, mice were assigned randomly to an EcN, EcN@SH or control treatment.
Blinding	The experimenters were blinded to the grouping information during data collection and analysis.

Reporting for specific materials, systems and methods

We require information from authors about some types of materials, experimental systems and methods used in many studies. Here, indicate whether each material, system or method listed is relevant to your study. If you are not sure if a list item applies to your research, read the appropriate section before selecting a response.

Materials & experimental systems

n/a	Involved in the study	
☐	☒	Antibodies
☒	☐	Eukaryotic cell lines
☒	☐	Palaeontology and archaeology
☐	☒	Animals and other organisms
☒	☐	Clinical data
☒	☐	Dual use research of concern

Methods

n/a	Involved in the study	
☒	☐	ChIP-seq
☐	☒	Flow cytometry
☒	☐	MRI-based neuroimaging

Antibodies

Antibodies used	Primary antibodies used include anti-rabbit ZO-1 (1:300; AF5145, Affinity), anti-mouse occludin (1:300; sc-133256, Santa Cruz); Secondary antibodies: goat anti-rabbit IgG (Alexa Fluor 594, 1:500; ab150080, abcam) and goat anti-mouse IgG (Alexa Fluor 488, 1:500; ab150113, abcam).
Validation	All used primary antibodies are commercially available and validated before.<br>Anti-rabbit ZO-1 (AF5145, Affinity) is validated for IF by the manufacturer and cited in 81 refs as reported on the manufacturer website (https://www.affbiotech.cn/goods-4452-AF5145-ZO_1_Antibody.html).

Anti-mouse Occludin (sc-133256, Santa Cruz) is validated for IF by the manufacturer and cited in 118 refs as reported on the manufacturer website (https://www.scbt.com/zh/p/occludin-antibody-e-5).

Animals and other research organisms

Policy information about studies involving animals; ARRIVE guidelines recommended for reporting animal research, and Sex and Gender in Research

Laboratory animals	6-8 weeks old ICR mice and 6-8 weeks old BALB/c mice (from Jiesijie Laboratory Animal Center) under controlled conditions (12:12-h light:dark cycle, room temperature 18-23 °C with 40-60% humidity).
Wild animals	The study did not involve wild animals.
Reporting on sex	Female ICR mice and male BALB/c mice were used.
Field-collected samples	The study did not involve field-collected samples.
Ethics oversight	All the animal procedures complied with the guidelines of the Shanghai Medical Experimental Animal Care. Animal protocols were approved by the ethics committee of Institutional Animal Care and Use Committee of Shanghai Jiao Tong University.

Note that full information on the approval of the study protocol must also be provided in the manuscript.

Flow Cytometry

Plots

Confirm that:

- [x] The axis labels state the marker and fluorochrome used (e.g. CD4-FITC).
- [x] The axis scales are clearly visible. Include numbers along axes only for bottom left plot of group (a 'group' is an analysis of identical markers).
- [x] All plots are contour plots with outliers or pseudocolor plots.
- [x] A numerical value for number of cells or percentage (with statistics) is provided.

Methodology

Sample preparation	Native or thiolated bacteria were washed with PBS. Cells were further incubated with corresponding fluorochrome-conjugated mucin.
Instrument	Beckman CytoFlex
Software	cytExpert
Cell population abundance	At least 10,000 relevant events were acquired for all FACS analysis.
Gating strategy	In general, cells were first gated on FSC/SSC. Singlet cells were gated using FSC-A and SSC-A. Dead cells were then excluded and further gating was performed on the live cell population.

- [x] Tick this box to confirm that a figure exemplifying the gating strategy is provided in the Supplementary Information.
